# Impact of the COVID-19 pandemic on the detection and management of colorectal cancer in England: a population-based study

**DOI:** 10.1016/S2468-1253(21)00005-4

**Published:** 2021-01-15

**Authors:** Eva J A Morris, Raphael Goldacre, Enti Spata, Marion Mafham, Paul J Finan, Jon Shelton, Mike Richards, Katie Spencer, Jonathan Emberson, Sam Hollings, Paula Curnow, Dominic Gair, David Sebag-Montefiore, Chris Cunningham, Matthew D Rutter, Brian D Nicholson, Jem Rashbass, Martin Landray, Rory Collins, Barbara Casadei, Colin Baigent

**Affiliations:** a Clinical Trial Service Unit and Epidemiological Studies Unit, Nuffield Department of Population Health, University of Oxford, Oxford, UK; b Medical Research Council Population Health Research Unit, Nuffield Department of Population Health, University of Oxford, Oxford, UK; c Big Data Institute, University of Oxford, Oxford, UK; d Cancer Research UK, London, UK; e Leeds Teaching Hospitals NHS Trust, Leeds, UK; f Leeds Institute for Medical Research at St James's, University of Leeds, Leeds, UK; g Leeds Institute of Health Sciences, University of Leeds, Leeds, UK; h NHS Digital, Leeds, UK; i Department of Colorectal Surgery, Oxford University Hospitals, Oxford, UK; j Population Health Sciences Institute, University of Newcastle, Newcastle, UK; k Department of Gastroenterology, North Tees University Hospital NHS Trust, Stockton on Tees, UK; l Nuffield Department of Primary Care Health Sciences, University of Oxford, Oxford, UK; m Department of Cardiovascular Medicine, John Radcliffe Hospital, Oxford, UK

## Abstract

**Background:**

There are concerns that the COVID-19 pandemic has had a negative effect on cancer care but there is little direct evidence to quantify any effect. This study aims to investigate the impact of the COVID-19 pandemic on the detection and management of colorectal cancer in England.

**Methods:**

Data were extracted from four population-based datasets spanning NHS England (the National Cancer Cancer Waiting Time Monitoring, Monthly Diagnostic, Secondary Uses Service Admitted Patient Care and the National Radiotherapy datasets) for all referrals, colonoscopies, surgical procedures, and courses of rectal radiotherapy from Jan 1, 2019, to Oct 31, 2020, related to colorectal cancer in England. Differences in patterns of care were investigated between 2019 and 2020. Percentage reductions in monthly numbers and proportions were calculated.

**Findings:**

As compared to the monthly average in 2019, in April, 2020, there was a 63% (95% CI 53–71) reduction (from 36 274 to 13 440) in the monthly number of 2-week referrals for suspected cancer and a 92% (95% CI 89–95) reduction in the number of colonoscopies (from 46 441 to 3484). Numbers had just recovered by October, 2020. This resulted in a 22% (95% CI 8–34) relative reduction in the number of cases referred for treatment (from a monthly average of 2781 in 2019 to 2158 referrals in April, 2020). By October, 2020, the monthly rate had returned to 2019 levels but did not exceed it, suggesting that, from April to October, 2020, over 3500 fewer people had been diagnosed and treated for colorectal cancer in England than would have been expected. There was also a 31% (95% CI 19–42) relative reduction in the numbers receiving surgery in April, 2020, and a lower proportion of laparoscopic and a greater proportion of stoma-forming procedures, relative to the monthly average in 2019. By October, 2020, laparoscopic surgery and stoma rates were similar to 2019 levels. For rectal cancer, there was a 44% (95% CI 17–76) relative increase in the use of neoadjuvant radiotherapy in April, 2020, relative to the monthly average in 2019, due to greater use of short-course regimens. Although in June, 2020, there was a drop in the use of short-course regimens, rates remained above 2019 levels until October, 2020.

**Interpretation:**

The COVID-19 pandemic has led to a sustained reduction in the number of people referred, diagnosed, and treated for colorectal cancer. By October, 2020, achievement of care pathway targets had returned to 2019 levels, albeit with smaller volumes of patients and with modifications to usual practice. As pressure grows in the NHS due to the second wave of COVID-19, urgent action is needed to address the growing burden of undetected and untreated colorectal cancer in England.

**Funding:**

Cancer Research UK, the Medical Research Council, Public Health England, Health Data Research UK, NHS Digital, and the National Institute for Health Research Oxford Biomedical Research Centre.

## Introduction

There is significant concern that the reorganisation of the National Health Service (NHS) in the UK in response to the COVID-19 pandemic has had a strongly negative effect on the management and outcomes of cancer.^1^, ^2^ Modelling studies have heightened fears of significant collateral damage^2^, ^3^, ^4^ but, to date, there has been little near-real-time, population-based evidence to substantiate these concerns. Such evidence is urgently required to inform services and to prevent those with cancer becoming unintended casualties of the COVID-19 pandemic.

The need is particularly urgent for colorectal cancer,^5^ where the impact of COVID-19 is likely to be substantial. The best outcomes are attained in those whose tumours are diagnosed at an early stage^2^, ^6^ but, unfortunately, the majority of the diagnostic and treatment pathways used in the management of the illness have been severely affected.^7^, ^8^, ^9^, ^10^ The initial phases of the COVID-19 service reorganisation led to the NHS Bowel Cancer Screening Programme being paused, and the main diagnostic tests of colonoscopy and CT colonography being limited to the emergency setting. In addition, significant changes were recommended to the gold standard treatment pathways in surgery, radiotherapy, and chemotherapy.^9^, ^10^, ^11^, ^12^ It is likely that many patients will, therefore, be experiencing a delay in both diagnosis and treatment.

**Research in context**

**Evidence before this study**

There is little evidence to quantify what effect the radical reorganisation of the UK National Health Service (NHS) in response to the COVID-19 pandemic has had on the management and outcome of colorectal cancer. There is significant concern, however, that the impact has been substantial. A search of PubMed using the search terms of “colorectal cancer” and “COVID-19” identified only modelling studies, surveys of care, and audits or case reports from individual hospitals. Modelling studies have predicted an increase in cancer-related deaths of over 15% due to both delays in diagnosis and difficulties in accessing treatment. Surveys and audits have suggested deviations from standards of care throughout the management pathway. No direct population-based evidence of the impact of COVID-19 on colorectal cancer care was identified. Given this disease is diagnosed in over 42 000 people and is the second biggest cancer-related cause of death in the UK each year it is already a major public health problem. Maintaining services is vital if we are, therefore, to minimise both the direct and indirect harms of COVID-19 on our population.

**Added value of this study**

This study provides quantitative information about the time course of changes in the diagnosis and management of colorectal cancer in NHS England during the COVID-19 pandemic. It demonstrates that the diagnostic pathway has been severely disrupted, with rapid reductions in urgent 2-week referrals and the use of colonoscopy, the main diagnostic test. In consequence, there has been a 22% reduction in the number of people being diagnosed and referred for first-line treatment on the 31-day pathway. As restrictions in the first lockdown were eased there was some recovery in both referrals and colonoscopies but not in the numbers entering the 31-day pathway. By October, 2020, rates had not exceeded 2019 levels, suggesting that, from April to October, 2020, over 3500 fewer people have been diagnosed and treated for colorectal cancer in England than would have been expected.

There were also major changes in patterns of care. In April, 2020, the number of surgical operations, the main curative treatment, fell by 31%. Over the summer, numbers slowly recovered but, by October, remained below 2019 levels. Surgical practice also changed, albeit in line with COVID-19 guidance, with a reduction in the proportion of people undergoing laparoscopic procedures and increases in the proportions of cases that formed a stoma and followed an emergency admission. The drop in the number of operations for rectal cancer was offset by an increase in the use of short-course radiotherapy (which can be used as a first-line treatment with and, in a minority of cases, without surgery).

**Implications of all the available evidence**

The reorganisation of the NHS in response to the COVID-19 pandemic has dramatically affected colorectal cancer services. Across England, there has been a major and sustained reduction in the detection of new colorectal cancers. Specialist services have been affected but, via changes in both surgical and oncological practice, they have adapted. As a second surge in COVID-19 cases is affecting the UK, and as new lockdowns are imposed, urgent action is required to overcome the barriers to diagnosis, as well as to protect the quality of service provision.

NHS leaders are actively seeking to adapt, restore, and maintain services but, to do that effectively, they need timely evidence on recent trends in service provision to inform their interventions. Ordinarily, official UK cancer statistics depend on the population-based incidence and treatment data captured by the UK's cancer registries. The collation of these high-quality registration datasets is, however, a relatively protracted process resulting in around 18 months' delay in full case ascertainment and official reporting. In the context of the COVID-19 pandemic, therefore, it is not possible to use these registry data to gain a timely population-based perspective on patterns of care. The novel use of other administrative health datasets may offer an alternative. For example, in the cardiovascular setting, the rapidly reported Secondary Uses Service Admitted Patient Care (SUSAPC) dataset^13^ has been used to quantify changes in presentation and care for those with acute coronary syndromes during the pandemic.^14^ The present study used analogous methods to compare care in 2019 with that in the first 10 months of 2020, including the periods before COVID-19, the first spring spike and lockdown, through to the emergence of the second wave. Future analyses will continue to track patterns of care until the challenges associated with the pandemic diminish.

## Methods

Information was extracted from four population-based datasets spanning NHS England over the time period Jan 1, 2019, to Oct 31, 2020: the National Cancer Waiting Time Monitoring Dataset (NCWTMD),^15^ the monthly diagnostics data (DM01),^16^ SUSAPC,^13^ and the National Radiotherapy Dataset (RTDS). Full details of the variables extracted from each dataset are described in the appendix (pp 2–5) but in brief, data were taken from the NCWTMD^15^ to investigate the number of people urgently referred for investigation with symptoms suggestive of lower gastrointestinal cancers (the 2-week wait—ie, the expected timeframe for referral from primary care for urgent specialist evaluation and investigation of individuals with red flag symptoms suggestive of a specific cancer type via pathways established in the UK) and the proportion seen within the target time. Colonoscopy is the main investigative test used to confirm a diagnosis of colorectal cancer. As such, the DM01 dataset was used to determine the total number of colonoscopies undertaken each month in NHS England over the study period.^16^ Once a person has a diagnosis of colorectal cancer they are referred for definitive treatment (surgery for colon cancer and surgery with or without neoadjuvant radiotherapy in rectal cancer) and this is expected to begin within 31 days of the decision to treat (the 31-day standard). Information was again extracted from the NCWTMD on the number of people with a diagnosis of a lower gastrointestinal cancer referred on this pathway and treated within the target timeframe.

To investigate the impact on treatment, information was extracted from the SUSAPC dataset on all individuals who underwent surgery in NHS England. These episodes of care were identified by examining all admissions that included a diagnosis of colorectal cancer defined using ICD-10 codes for colonic (C18) and rectal (C19/C20) cancer (appendix p 7). The first admission in which each individual underwent a major surgical procedure (either a major resection or non-resectional operation [a stoma or a bypass]) defined using the Office of Population Censuses and Surveys Classification of Surgical Operations and Procedures 4th revision^17^ codes (appendix pp 7–10) was then extracted. Patterns of use of surgery were then investigated both overall and, as the management pathways for colonic and rectal cancer differ, by tumour site.

As part of the COVID-19 response, the NHS and several relevant professional bodies offered guidance on changes to standard surgical practice.^9^, ^10^, ^12^, ^18^ For example, laparoscopic and robotic surgery (used routinely in colorectal surgery) are aerosol-generating procedures so may increase the risk of exposure to SARS-CoV-2. As such, initial guidance recommended surgery should be undertaken via an open abdominal procedure.^10^ Similarly, to reduce the risk of anastomotic leaks (a serious complication of colorectal cancer surgery which often necessitates a patient returning to theatre or being admitted to critical care) it was recommended that consideration should be given to the formation of stomas in preference to performing an anastomosis or using such a procedure to cover an anastomosis.^12^ Rates of use of laparoscopic surgery and the use of stoma forming operations (following both elective and emergency admissions) were, therefore, also investigated.

A substantial proportion of patients with colorectal cancer (mainly those with colonic cancer) present as an emergency.^19^ With increases in the time to diagnosis anticipated during the pandemic there may be a concomitant increase in the proportion of cases treated urgently. As such, any change in the proportion of operations undertaken following an emergency admission was investigated.

In rectal cancer, definitive treatment can include the use of neoadjuvant radiotherapy. In April, 2020, international consensus guidance^11^ published in response to COVID-19 advised that short-course radiotherapy with a delay to surgery could be used to minimise the risk of individuals being exposed to SARS-CoV-2. To investigate trends in use of radiotherapy, information was extracted from RTDS on all radical treatments administered to rectal tumours (ICD-10 code C20). Courses were categorised (appendix p 4) into long-course radiotherapy, short-course radiotherapy, and other prescriptions.

As a result of the major pressures in hospitals resulting from COVID-19, it was recognised that there may have been a reduction in the speed and completeness of clinical coding within both the SUSAPC and RTDS datasets resulting in artifactual declines in treatment rates (particularly in the most recent data). A robust, recently developed method^14^ was used to address this. For each month from March, 2020, the proportion of all episodes recorded in SUSAPC that contained no diagnostic ICD-10 codes was determined and, based on these figures, a subsequent monthly adjustment was made to the numbers of recorded admissions with colorectal cancer. In the RTDS, a number of radiotherapy centres had not submitted data on the treatments they had delivered in June to October, 2020. The radiotherapy figures were increased by the proportion of cases usually submitted by these centres, resulting in an upward adjustment of about 4% for October, 2020, and 1% or less for earlier months.

### Statistical analysis

In all analyses examining trends in the numbers of admissions, as well as of surgical and radiotherapy treatments, data are presented as the monthly number of events reported in 2019 and the adjusted monthly figures for 2020 with a connected line graph fitted through these values and error bars representing plus or minus 1 SD of the pre-COVID-19 2019 monthly counts. Figures derived from the SUSAPC and RTDS datasets for 2020 have been adjusted for missing data (appendix p 5). Analyses examining stoma use, laparoscopic surgery, and operations following an emergency admission, compare trends in the proportion of cases affected. Percentage changes in monthly figures were calculated by comparing the adjusted monthly number for the relevant month in 2020 with the mean monthly number during 2019; these changes are shown with 95% CIs based on the ratio of two rates (assuming monthly counts follow a Poisson distribution and after correction for over-dispersion). Analyses were undertaken using Stata 16.

### Role of the funding source

Cancer Research UK were involved in data analysis, data interpretation, and the writing of the report. Public Health England and NHS Digital were involved in data collection, data analysis, data interpretation, and the writing of the report. The other funders had no role in study design, data collection, data analysis, data interpretation, or writing of the report. All authors had full access to the data and the corresponding author had the final responsibility to submit for publication.

## Results

**Figure 1:**
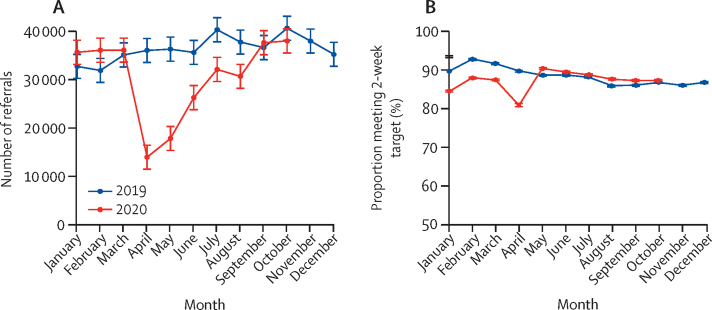
Monthly number of referrals into the 2-week wait pathway (A) and the proportion meeting 2-week target in England (B) Error bars represent +/– 1 SD of the (pre-COVID-19) monthly counts for 2019.

In England during 2019, there were a mean of 36 274 (SD 2663) referrals per month into the 2-week pathway. Beyond March, 2020, when the first lockdown began, patterns of referral for suspected cancer changed radically (table 1, figure 1), so that in April, 2020, there were 13 440 2-week wait referrals, representing a 63% (95% CI 53–71) relative reduction compared with the monthly average in 2019 (table 1, figure 1). Over subsequent months there was a gradual recovery, and by October, 2020, 2-week referral rates had returned to 2019 levels. There was also a sudden reduction in the proportion of referrals meeting the 2-week target: during 2019 a mean of 88% (SD 2) of referred patients were seen within 2 weeks, but this fell to 81% in April, 2020, before recovering rapidly (table 1, figure 1).

**Table 1:** Monthly number, and percent reduction in the number, of referrals per standard cancer waiting times, and the number within the target time period, for lower gastrointestinal cancers* in England

	**2-week wait referrals**				**31-day to treatment referrals**			
	All referrals		Number seen within target		All referrals		Number seen within target	
	n	Percent reduction (95% CI)	n	Percent reduction (95% CI)	n	Percent reduction (95% CI)	n	Percent reduction (95% CI)
2019 monthly mean (SD)	36 274 (2663)	..	31 938 (1909)	..	2781 (205)	..	2684 (198)	..
January, 2020	35 563	2% (−14 to 16)	29 917	6% (−6 to 17)	3035	−9% (−26 to 6)	2845	−6% (−23 to 8)
February, 2020	35 992	1% (−15 to 15)	31 586	1% (−12 to 13)	2672	4% (−12 to 18)	2575	4% (−12 to 18)
March, 2020	36 014	1% (−15 to 15)	31 371	2% (−11 to 13)	3068	−10% (−27 to 4)	2962	−10% (−27 to 4)
April, 2020	13 440	63% (53 to 71)	10 822	66% (58 to 72)	2158	22% (8 to 34)	2074	23% (8 to 35)
May, 2020	17 375	52% (41 to 61)	15 665	51% (42 to 59)	1802	35% (22 to 46)	1630	39% (27 to 50)
June, 2020	26 000	28% (15 to 40)	23 197	27% (16 to 37)	1932	30% (17 to 42)	1788	33% (20 to 44)
July, 2020	31 944	12% (−3 to 25)	28 268	11% (−1 to 22)	2168	22% (8 to 34)	2067	23% (9 to 35)
August, 2020	30 474	16% (1 to 29)	26 609	17% (5 to 27)	2136	23% (9 to 35)	2023	25% (11 to 37)
September, 2020	37 587	−4% (−20 to 11)	32 525	−2% (−15 to 10)	2663	4% (−12 to 18)	2482	8% (−8 to 21)
October, 2020	37 952	−5% (−21 to 10)	33 389	−5% (−18 to 7)	2824	−2% (−18 to 13)	2686	0% (−16 to 14)
April to October, 2020, monthly mean (SD)	27 825 (9498)	23% (17 to 29)	24 354 (8453)	24% (19 to 28)	2240 (372)	19% (13 to 25)	2107 (369)	22% (15 to 27)

* May include suspected anal cancers.

**Figure 2:**
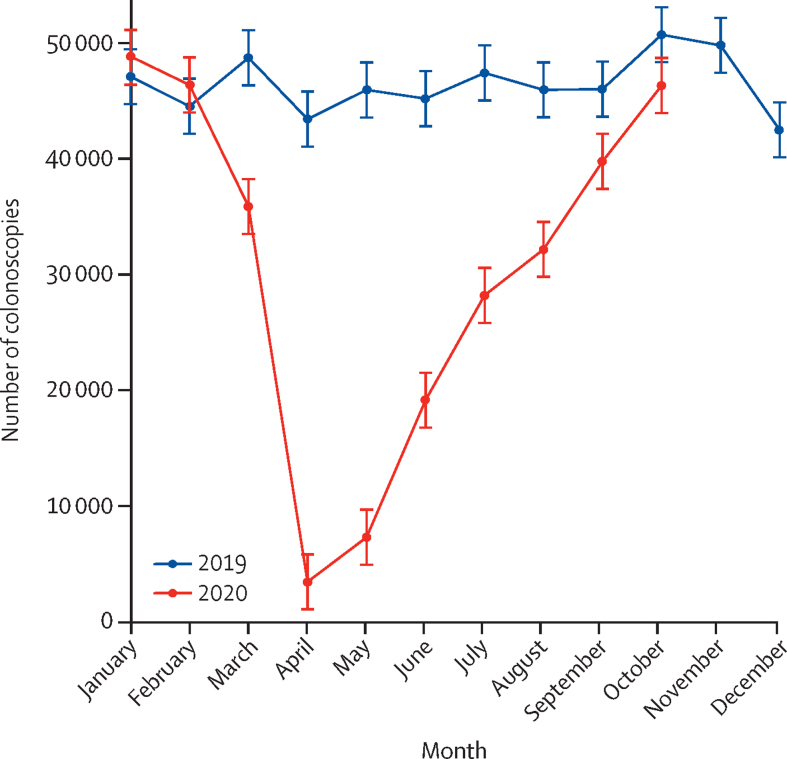
Monthly number of colonoscopies undertaken in England Error bars represent +/– 1 SD of the (pre-COVID-19) monthly counts for 2019.

During 2019, a mean of 46 441 colonoscopies (SD 2456) were performed monthly (table 2). In April, 2020, there were just 3484 colonoscopies performed in England, representing a 92% (95% CI 89–95) relative reduction compared with the monthly average in 2019. In subsequent months, there was a gradual recovery such that by October, 2020, rates had returned to the 2019 monthly average (figure 2, table 2).

**Table 2:** Monthly number, and percent reduction in the monthly number, of colonoscopies and operations undertaken in England

	**n**	**Percent reduction (95% CI)**
**Colonoscopies**		
2019 monthly mean (SD)	46 441 (2456)	..
January, 2020	48 804	−5% (−17 to 5)
February, 2020	46 344	0% (−11 to 10)
March, 2020	35 851	23% (13 to 32)
April, 2020	3484	92% (89 to 95)
May, 2020	7332	84% (79 to 88)
June, 2020	19 155	59% (51 to 65)
July, 2020	28 201	39% (30 to 47)
August, 2020	32 163	31% (21 to 39)
September, 2020	39 752	14% (4 to 24)
October, 2020	46 295	0% (−11 to 10)
April to October, 2020, monthly mean (SD)	25 197 (16 029)	46% (42 to 49)
Operations*		
2019 monthly mean (SD)	2003 (136)	..
January, 2020	2245	−12% (−28 to 2)
February, 2020	1979	1% (−14 to 14)
March, 2020	2129	−6% (−22 to 7)
April, 2020	1378	31% (19 to 42)
May, 2020	1339	33% (21 to 43)
June, 2020	1576	21% (8 to 33)
July, 2020	1712	15% (1 to 26)
August, 2020	1662	17% (3 to 29)
September, 2020	1913	4% (−10 to 17)
October, 2020	1859	7% (−7 to 20)
April to October, 2020, monthly mean (SD)	1634 (220)	18% (13 to 24)

* Numbers between March and October, 2020, are adjusted using the methods described in the appendix (p 4) for any reduction in coding completeness.

**Figure 3:**
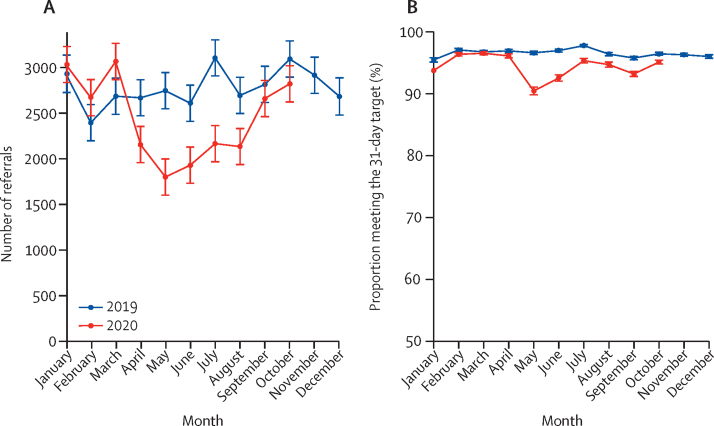
Monthly number of 31-day to treatment pathway referrals (A) and the proportion of referrals meeting that target in England (B) Error bars represent +/– 1 SD of the (pre-COVID-19) monthly counts for 2019.

A mean of 2781 (SD 205) individuals with a confirmed diagnosis of colorectal cancer entered the 31-day-to-treatment pathway per month in 2019 (table 1). In April, 2020, there were 2158 referrals, representing a 22% (95% CI 8–34) relative reduction compared with the monthly average in 2019 (table 1, figure 3). There was further decline in May, before a gradual recovery and by October the monthly number of referrals had returned to 2019 levels (table 1, figure 3). Given there is no evidence that the incidence of cancer has changed, this suggests that there are more than 3500 fewer cases diagnosed between April and October, 2020, than would have been expected based on 2019 referrals. During 2019, an average of 96% of those referred into this pathway met the 31-day target: the proportion fell slightly after May, 2020, but had recovered to 95% by October, 2020 (table 1, figure 3).

**Figure 4:**
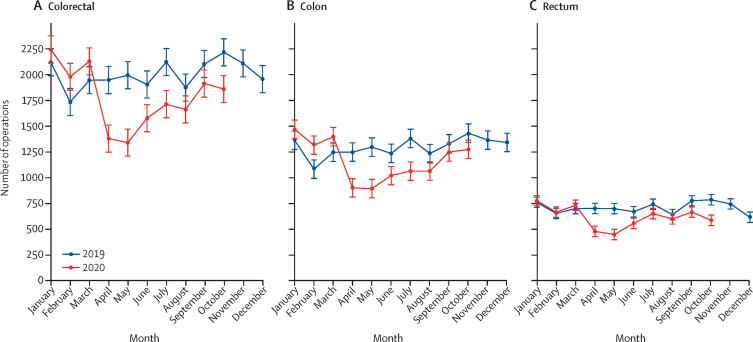
Monthly number of operations undertaken for (A) colorectal, (B) colonic, and (C) rectal cancer in England Error bars represent +/– 1 SD of the (pre-COVID-19) monthly counts for 2019. The 2020 rate is based on counts adjusted for incomplete coding (appendix p 4).

During 2019 a mean of 2003 (SD 136) colorectal cancer operations were performed monthly in England. In April, 2020, there were 1378 such operations, representing a relative reduction of 31% (95% CI 19–42) overall (with a 31% [95% CI 17–42] reduction for colon and a 32% [18–44] reduction for rectum) compared with the monthly mean in 2019 (table 2). Numbers remained low in May and began to recover in June (table 2, figure 4) but, by October, they remained below the 2019 monthly average.

Amongst the reduced number of operations that were undertaken, surgical practice changed markedly. During 2019, a mean of 1175 (SD 95) of 2003 colorectal cancer operations, or 59% (SD 2) were undertaken laparoscopically, but in April, 2020, this proportion fell to 25%. Over the summer this proportion recovered such that, by October, 2020, this proportion was 61% (appendix pp 12–17).

During 2019, the mean proportion of colorectal cancer operations involving stoma formation was 44% (SD 2), with no evidence of seasonal variation. A higher proportion of operations for rectal cancer (80% [SD 2]) resulted in stoma formation than for colon cancer (25% [2]; appendix pp 12–14, 16). After April, 2020, the proportion of colorectal operations resulting in a stoma increased to 56% (83% rectal, 42% colon), and remained above the 2019 monthly average.

During 2019 the average proportion of colorectal operations that followed an emergency admission was 20% (SD 9). Between April and August, 2020, there was a small, but sustained, increase in this proportion (appendix pp 12–14, 16) but by October, 2020, it had returned to be equivalent to 2019 monthly levels.

**Figure 5:**
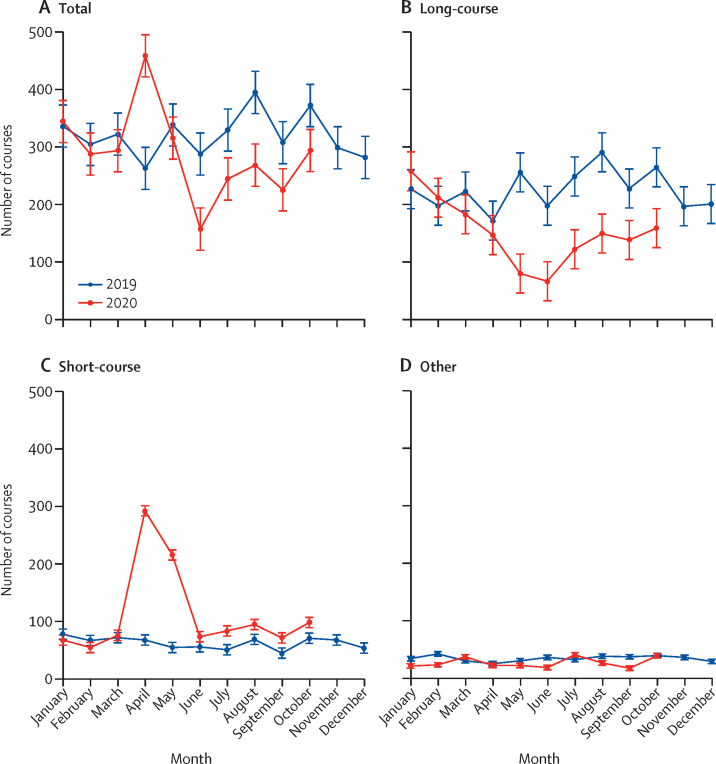
Monthly number of episodes (courses) of neoadjuvant rectal radiotherapy delivered, by treatment type, in England Error bars represent +/– 1 SD of the (pre-COVID-19) monthly counts for 2019. The 2020 rate is based on counts adjusted for incomplete coding (appendix p 5).

There was a mean of 321 (SD 38) courses of radiotherapy for rectal cancer delivered monthly in England in 2019 (table 3). In April, 2020, this increased to 461 courses, representing a 44% (95% CI 17–76) relative increase (table 3), before falling by July to below the 2019 monthly average (figure 5). This pattern, however, was determined by different trends in the type of radiotherapy delivered. In 2019, the average monthly proportion of radiotherapy that was in a long-course prescription was 70% (SD 4) whereas 19% (SD 3) was short-course. In April, 2020, the monthly number of long-course treatments fell sharply and represented only 32% of treatments delivered. By contrast, the number of short-course episodes almost quadrupled and represented 63% of radiotherapy treatments delivered. In subsequent months until October, 2020, the use of long-course radiotherapy remained low and the use of short-course remained above 2019 levels (table 3).

**Table 3:** Monthly number, and percent reduction in the monthly number, of episodes (courses) of neoadjuvant rectal radiotherapy delivered in England

	**Total**		**Long-course**		**Short-course**		**Other**	
	n*	Percent reduction (95% CI)	n*	Percent reduction (95% CI)	n*	Percent reduction (95% CI)	n*	Percent reduction (95% CI)
2019 monthly mean (SD)	321 (38)	..	225 (35)	..	62 (10)	..	34 (5)	..
January, 2020	346	−8% (−36 to 15)	258	−14% (−54 to 15)	67	−8% (−50 to 22)	21	38% (11 to 57)
February, 2020	289	10% (−16 to 30)	212	6% (−30 to 32)	54	13% (−25 to 39)	23	32% (4 to 52)
March, 2020	295	8% (−18 to, 29)	183	19% (−15 to 42)	75	−21% (−65 to 11)	37	−9% (−44 to 18)
April, 2020	461	−44% (−76 to −17)	147	35% (4 to 55)	292	−372% (−462 to −296)	22	35% (7 to 55)
May, 2020	317	1% (−26 to 23)	80	65% (41 to 79)	215	−248% (−323 to −186)	22	35% (7 to 55)
June, 2020	158	51% (31 to 65)	67	70% (48 to 83)	73	−18% (−60 to 14)	18	47% (21 to 64)
July, 2020	245	24% (0 to 42)	122	46% (18 to 64)	83	−34% (−80 to 0)	40	−19% (−56 to 9)
August, 2020	270	16% (−9 to 35)	149	34% (3 to 55)	94	−52% (−100 to −15)	26	23% (−8 to 45)
September, 2020	226	30% (6 to 47)	138	39% (9 to 59)	71	−14% (−57 to 17)	17	50% (24 to 66)
October, 2020	295	8% (−18 to 28)	159	29% (−2 to 51)	97	−57% (−107 to −20)	39	−14% (−50 to 14)
April to October, 2020, monthly mean (SD)	282 (94)	12% (2 to 22)	123 (36)	45% (35 to 54)	132 (86)	−114% (−142 to −89)	26 (9)	22% (10 to 33)

* Numbers in 2020 are adjusted using the methods described in the appendix (p 5) for any reduction in submission of data.

## Discussion

This is the first study to assess, in near real time, the impact of the COVID-19 pandemic on the management of colorectal cancer across England. It has shown marked changes across the pathway of care. The number of people being referred to hospital with suspected lower gastrointestinal cancer, and the number subsequently diagnosed with the disease, fell sharply during the first lockdown and a deficit persisted up until September, 2020. This translates to over 3500 fewer people than expected beginning treatment between April and October, 2020. Survival from colorectal cancer is closely linked to stage of disease, with over 90% of those diagnosed at stage I surviving 5 years compared with only 10% at stage IV.^20^ As delays in diagnosis allow tumours to continue to grow and advance,^3^, ^21^ this bottleneck in the diagnostic pathway is likely to have a profoundly detrimental impact on colorectal cancer outcomes in England.^6^

Before steps can be taken to address this reduction in the number of confirmed diagnoses of colorectal cancer, it is necessary to understand why it has arisen. Evidence suggests the lockdown led to a 30% reduction in primary care consultations^22^ with 50–60% of consultations being conducted remotely. Whether this was a result of patient concern about accessing primary care or difficulties in access due to restrictions on services is unclear. Similarly, shielded symptomatic patients consulting primary care remotely may have chosen to be monitored at home in preference to being referred to a specialist. General practitioners were advised to limit, and in some cases were prevented from making, referrals in an effort to protect secondary care capacity, resulting in a 50–74% reduction in referral rates.^22^ Challenges in undertaking colonoscopy, the main diagnostic test for colorectal cancer, in a COVID-secure way may also be a factor.^23^, ^24^ In response, faecal immunochemical testing was variably introduced to triage symptomatic patients for referral from primary care and to prioritise referrals in secondary care. Further research is required to better understand the role of these contributory factors in determining why fewer patients are presenting in hospital. With the incidence of COVID-19 now rising rapidly again, in addition to the normal winter health-care strains, this recovery will be a major challenge. Our analyses provide evidence to help monitor trends and so inform operational policy and will be revised each month and published online^25^ to help inform services as the pandemic evolves.

Amongst those who did receive a diagnosis of colorectal cancer, there were major changes in the delivery of treatment. The number of operations fell sharply and surgical methods were adapted to minimise COVID-19 risk. While these changes may have helped protect patients from SARS-CoV-2, and were in line with guidance written in response to the pandemic, they are deviations from accepted standards of care. While some consequences of these changes are predictable, the long-term impact of others is uncertain. For example, the proportion of people receiving a stoma increased and, based on the number of procedures performed, our analyses suggest that an additional 600 people have received a stoma than would have been expected. In many of these cases, the stoma will have been intended as a temporary measure until it could be reversed to restore gastrointestinal continuity. Given stomas often have a detrimental impact on long-term quality of life,^26^ and additional care costs, such reversals will be important. However, as there is already a deficit in the number of operations undertaken, as well as renewed and growing pressure on NHS services as the number of COVID-19 cases increases again, scheduling these additional operations may prove to be a challenge.

The opposite trend was seen with laparoscopic surgical procedures with, in April, a steep decline in the proportion in whom they were used. Again, there was a gradual recovery, but rates remain lower than 2019 levels. Given that they are a standard treatment in colorectal cancer, associated with faster postoperative recovery times and reduced hospital stays, they are the preference of many and a recommended standard of care. Restoring safe laparoscopic colorectal cancer services is, therefore, important.

The increase in the proportion of operations that followed an emergency admission may heighten concerns about the impact of COVID-19 on cancer outcomes. Individuals who present urgently have a significantly worse prognosis than those presenting electively^27^ and with standard colorectal cancer diagnostic pathways being severely disrupted^23^ these increases may be an early indication of diagnostic and treatment delays creating a stage-shift at presentation. Although the change observed is small and may be influenced by differences in the coding of admission types during the pandemic, the small increase seen could be an early signal, also seen internationally,^28^ of the impact of delayed diagnoses. It is important, therefore, to continue to monitor the number and proportion of admissions that were emergencies and ensure the trend is reversed.

In rectal cancer, a decrease in the number of operations occurred in parallel with a significant increase in the use of radiotherapy, predominantly in the form of short-course radiotherapy. The rapid increase in its use is likely to reflect the use of short-course radiotherapy for patients with early stage disease who would not normally receive preoperative radiotherapy and a preference for short-course over long-course treatment in patients where preoperative radiotherapy is normally indicated. For patients with early stage disease who would not ordinarily have received it, radiotherapy may be acting as a bridge to surgery, with individuals receiving it as a holding treatment. Although an effective therapy to reduce local recurrence, radiotherapy is also associated with greater morbidity when combined with radical surgery and does not influence survival,^29^ so its use may have a detrimental impact on quality of life. By contrast, a substantial minority (depending on case mix) may attain a complete clinical response, allowing an active surveillance approach and the avoidance of surgery altogether (the concept of organ preservation). Although recent guidelines from the National Institute of Health and Care Excellence recommended that radiotherapy should not be used in early stage rectal cancer outside of clinical trials, it is likely that this approach was used during the first wave peak of COVID-19 in some patients. Given the scale of the change in practice observed, the potential consequences for the rectal cancer population are significant. It is vital, therefore, that the outcomes of this population are closely monitored as their results may have long-term implications on the use of radiotherapy in the management of rectal cancer.^30^

Although this study provides timely information, the data it is based upon have some limitations. For example, some NHS Trusts may have submitted data to the SUSAPC without a diagnostic code, leading to potential under-ascertainment. This limitation has been addressed by applying an adjustment (developed and validated in a previous study^14^), which has been demonstrated to mitigate any such bias. Another limitation is that not all colorectal cancers are captured within the datasets used and some cases will be miscoded. Efforts are underway to create a more timely rapid registration dataset^31^ to inform care during the COVID-19 period. This will, however, rely on similar rapid data feeds to those used in this study so will experience similar data quality and case ascertainment issues. A more realistic rapid alternative would be to take data directly from the electronic patient record in each hospital (such as in the NIHR Colorectal Cancer Health Informatics Collaborative).^32^ Unfortunately, data flows within the NHS are not yet sufficiently mature to enable this to happen at population level and, in consequence, although this study provides the most timely data available, they are not timely enough. There are other limitations that need to be addressed. For example, data could not be linked at a person level across the datasets involved, preventing adequate assessment of any trends in relation to important demographic characteristics such as age, ethnicity, or socioeconomic status. It was also not possible to access all the datasets at a provider or regional level, preventing more detailed geographical analyses of care which are important in assessing how COVID-19 rates in the community and the varying tiers of restrictions have impacted on care. Key aspects of the management pathway could also not be examined because the data needed were not sufficiently rapidly available. Linkage to primary care data would have enabled the role of changes in pre-referral consultations to be investigated and it was also not possible to rapidly access information on the important modality of chemotherapy. This is an important treatment, primarily used as an adjuvant treatment and in the management of metastatic disease. As a result of the FOxTROT trial,^33^ there may also be growing use in the neoadjuvant setting. As our focus in this paper was on the main first-line, potentially curative treatments, we chose not to wait for data to become available to investigate these aspects of the care. Such work is, however, urgently needed so the full colorectal cancer pathway can be assessed.

Our inability to monitor such patterns of care in real time prevents corrective action being taken when problems arise rather than when they have become established. Furthermore, even where datasets are available, the essential restrictions in place to protect patient confidentiality can make obtaining the necessary permissions to access and link the required data an extremely lengthy process. Revisions to systems to enable faster data acquisition and access for applied research that informs care, while also respecting patient confidentiality, are, therefore, required.^34^ Given the striking reduction in 2-week wait referrals and, in consequence, the decrease in new diagnoses entering the 31-day pathway that this study has shown, there is now sufficient evidence to justify immediate action. Eliminating and overcoming these diagnostic delays is vital to prevent further harm to the outcomes of those with colorectal cancer.

When these data mature and other resources become available, it will be of paramount importance to investigate surgical outcomes such as postoperative mortality, returns to theatre, margin involvement, lymph node yield, and stoma reversal rates, as well as complete response rates in those given rectal radiotherapy, post-treatment morbidity, and long-term survival. Given that our study has clearly demonstrated significant delays in diagnosis, any change in stage at presentation will also be vitally important to monitor as an early surrogate for the long-term impact on prognosis. Such information is essential not only for individuals, but also to help ensure high quality services for all through the COVID-19 pandemic.

This study is the first to provide operational detail on what is happening to patients with colorectal cancer during the pandemic, rather than modelled estimates. The results are stark, stretch across the full patient pathway, and are likely to have a significant impact both in terms of the prognosis of those diagnosed with the disease and management costs^35^ for the NHS. Early evidence suggests the trends may not be limited to the UK.^36^, ^37^, ^38^, ^39^, ^40^ These deficits in diagnosis and deviations from standard care pathways will continue to grow until services are restored and capacity allows the backlogs to be addressed. There is a growing body of evidence to suggest similar shortfalls in diagnosis and treatment may be observed internationally. Resolution will demand both campaigns to ensure the public continue to seek help if they develop symptoms suggestive of colorectal cancer, alongside resources to ensure colorectal cancer services are protected and maintained during the pandemic. Ongoing monitoring, through analysis of timely and synergised data sources, is required to ensure these challenges are addressed rapidly, with further analysis crucial to understanding their full impact on cancer outcomes.

## Data sharing

The data this study are based on are available on successful application to NHS Digital (SUSAPC), Public Health England (RTDS) or via the NHS England website (NCWTMD and DM01). The project team will continue to update the analyses based on these data and publish them (alongside any developments to the methods used) at http://www.ndph.ox.ac.uk/corectr/covid19.
