# Supplementary material for: Impact of the COVID-19 pandemic on the detection and management of colorectal cancer in England: a population-based study
Source: Lancet Gastroenterol Hepatol. 2021 Jan 15;6(3):199–208. doi: 10.1016/S2468-1253(21)00005-4 (PMC7808901; doi:10.1016/S2468-1253(21)00005-4)

THE LANCET
Gastroenterology & Hepatology

Supplementary appendix

This appendix formed part of the original submission and has been peer reviewed. We post it as supplied by the21 authors.

Supplement to: Morris EJA, Goldacre R, Spata E, et al. Impact of the COVID-19 pandemic on the detection and management of colorectal cancer in England: a population-based study. *Lancet Gastroenterol Hepatol* 2021; published online Jan 14. https://doi.org/10.1016/S2468-1253(21)00005-4.

Contents

Supplementary methods 2
Record selection and data extraction 2
Diagnostic Waiting Times and Activity Dataset 2
Record selection and data extraction 2
Secondary Uses Service Admitted Patient Care (SUSAPC) dataset 2
Record selection and data extraction 3
Data completeness and consistency in the SUSAPC data set 4
National Radiotherapy Dataset 5
Record selection and data extraction 5
Data completeness and consistency in the RTDS 5
Supplementary methods for subsequent analyses 6
References for supplementary methods 6
Supplementary Results 11

Supplementary methods

This section describes the methods used for the initial analyses conducted on data available up to 27 November 2020. It is intended these will be updated regularly using refreshed data and further data quality work will also continue in parallel. Any resulting methodological updates will be described at http://www.ndph.ox.ac.uk/corectr/covid19

Cancer Waiting Times

The National Cancer Waiting Times Monitoring Dataset is captured by the NHS to fulfil the requirement to enable performance monitoring of cancer services delivered by both healthcare providers and clinical commissioning groups in England. Services are monitored in relation to targets on waiting time laid out in National Guidance, currently 93% of patients referred under a Two Week Wait should be seen within this timeframe, and 96% of patients who are diagnosed with cancer should start their treatment within 31 days of the decision to treat the patient.[1] The results are openly published on both a quarterly and monthly basis.[1]

Record selection and data extraction

Monthly figures were taken from the provider reports[1] on both the number of people who attended outpatient appointments within two weeks of an urgent referral by their General Practitioner (GP) for a suspected lower gastrointestinal cancer and, for any patient with cancer regardless of referral route, the numbers who started their first definitive treatment within 31 days of decision to treat.

Diagnostic Waiting Times and Activity Dataset

The Diagnostics Waiting Time and Activity dataset (DM01)[2] captures information on the waiting times and frequency of 15 different diagnostic tests and procedures. It is openly reported and used by NHS England to measure performance against the diagnostic operation standard (which is that less than 1% of people should wait over 6 weeks for a diagnostic test). The dataset contains aggregated information submitted by providers and broken down at commissioner level examining the number of procedures undertaken, as well information around the waiting times targets.

Record selection and data extraction

Information was extracted from DM01 on all colonoscopies (the main diagnostic test for colorectal cancer) undertaken each month between January 1 2019 and October 31 2020 across England.[2]

Secondary Uses Service Admitted Patient Care (SUSAPC) dataset

The Secondary Uses Service Admitted Patient Care (SUSAPC) dataset[3] is a repository of data hosted by NHS Digital that relates to in-patient care provided in England, which aims to enable reporting and analyses to support the NHS in the delivery of healthcare services. These data are submitted on

a regular basis by NHS hospital trusts at pre-arranged dates during the year. Submissions are consolidated, validated and cleaned and then incorporated into the Hospital Episode Statistics (HES) dataset.[4] Analyses from SUSAPC are labelled as 'management information', a term used to describe aggregate information collated and used in the normal course of business to inform operational delivery, policy development or the management of organisational performance. This recognises that data may be incomplete in places and is not quality assured to the same extent as HES.

In the SUSAPC dataset, each record contains data relating to a continuous period of care under one consultant known as a Finished Consultant Episode (FCE). Each FCE contains data about the patient (e.g. date of birth, ethnicity), the specialty providing the care (e.g. surgery), the start and end date of the continuous period of care, the diagnoses reasons (captured using International Classification of Diseases 10th revision (ICD-10) codes[5]) and treatments delivered (recorded using Office of Population Censuses Surveys Classification of Surgical Operations and Procedures 4th revision (OPCS4) codes[6] along with dates they occurred on. For this analysis, if there are adjoining multiple FCEs for an individual then that have been combined to form a spell. A spell is a continuous period of in-patient care provided by a single hospital and, in this analysis is what constitutes an 'admission'.

Record selection and data extraction

All FCEs starting between October 1 2018 and October 31 2020 and containing an ICD-10 code indicating colorectal cancer in any diagnostic position were identified. All FCEs in the same spell were considered relevant and the following data were extracted: age at admission; ethnicity; date FCE started; date FCE finished; source of the admission; method of admission; health care provider; ICD-10 diagnostic codes; and OPCS-4 procedure codes.

All admissions were included in which a colorectal cancer was recorded as a diagnosis using the ICD10 codes C18-C20. Colon cancers were deemed to be those with a C18 code whereas rectal tumours were those with either a C19 or C20 code. If individuals had multiple codes within this range across a hospital admissions then they were classed as a C188 which represents a colon cancer with an overlapping site.

Surgical procedures were identified using the OPCS4 codes listed in supplementary Table 2. The first procedure each individual in the cohort underwent was used as a surrogate for initial surgical treatment. If individuals underwent multiple procedures in the same admission then the one delivered on the earliest date was selected. If individuals underwent multiple procedures on the same day then the most extensive procedure in relation the cancer site was selected.

Laparoscopic procedures were defined as those operations with an accompanying OPCS4 code listed in Table 3 on the same date as the code indicating the procedure.

Stoma forming operations were defined by those codes listed in Table 5 and Table 4 or any operation in Table 2 with one of the codes in Table 5 recorded on the same date.

Emergency admissions and procedures were defined as those in which the admission method was classified as an emergency (i.e. where the SUSAPC ADMIMETH field did not contain 11, 12 or 13).

Analyses are limited to data submitted by NHS hospital trusts identified as having one or more colorectal cancer multidisciplinary teams or independent hospitals delivering NHS funded care.

Data completeness and consistency in the SUSAPC data set

In order to obtain the most up-to-date information about admissions with colorectal cancer, and interpret findings across the entire time period of interest, the SUSAPC data were analysed to facilitate consistency in interpretation from the earliest to the latest reported period of interest. Underreporting of colorectal cancer diagnosis codes could mimic a real reduction in activity. Alternatively, such under-reporting could be the result of delayed submission of SUSAPC data to NHS Digital, incomplete coding of diagnoses within submitted FCEs, or incomplete FCEs at the time of the data freeze.

Most NHS hospital trusts submit SUSAPC data on a monthly basis by a particular deadline. This follows a two-phase reconciliation process to arrive at a final agreed position for each month's activity as defined in the NHS Standard Contract. In the first phase of reconciliation, providers submit initial calendar month activity data to SUS and a snapshot of these data is taken to a fixed national deadline. A second snapshot is taken at a deadline one month later. The data freeze for the initial analyses was after the first phase reconciliation deadline for October 2020 activity, and the second phase reconciliation deadline for September 2020 activity.

To assess any bias from reduced coding activity, the numbers of all FCEs for all hospitalisations submitted with no diagnostic ICD-10 codes each month were examined and a notional adjustment was made to the absolute numbers of admissions with colorectal cancer each month (adjusted number of admissions = observed number of admissions x total number of FCEs/(total number of FCEs - number of FCEs without diagnostic codes).

Data are only submitted when the FCE is completed (i.e. the patient has either died, been discharged, or been transferred to the care of another consultant) meaning that individuals who have been admitted but have not yet completed an FCE are not counted. As the median length of stay in which a major resection took place is 10 days this is likely to have affect case ascertainment in the data available for October and so surgical analyses are only presented up to September 2020.

National Radiotherapy Dataset

The Radiotherapy Data Set (RTDS) collates data from all NHS providers of radiotherapy services in England to provide consistent and comparable data on radiotherapy services in England. Data are collected for all radiotherapy in the forms of teletherapy and brachytherapy (including automated remote afterloading machines) for malignant disease delivered in England to patients in NHS facilities, or in private facilities where delivery is funded by the NHS.

Record selection and data extraction

Information was provided from the RTDS on the weekly number of new radiotherapy (excluding brachytherapy) episodes (defined as those with a new treatment start date) delivered in England within the period of Jan 1 2019 and Oct 31 2020. Only episodes in which the treatment intent was described as radical and the primary diagnosis was rectal cancer (ICD10 code C20) were included.

Episodes were then classed into long-course, short-course and other radiotherapy using the prescribed total dose and the prescribed number of fractions. Long-course radiotherapy included episodes that were 45-50.4 Gray (Gy) in 25-28 fractions, 45Gy in 20 fractions or 52-60Gy in 25 to 30 fractions. Short-course radiotherapy included episodes that were 25Gy in 5 fractions or 20Gy in 4-5 fractions. Other radiotherapy included all other remaining prescribed dose and fraction combinations and those where dose &/or fraction fields were missing.

These categorisations were based on planned prescribed dose and fraction schedules. Actual treatments delivered may have varied from these plans. Doses in Gy is given with a +/-1 tolerance to account for some adjustments when schedules were calculated. For example, a planned dose fractionation schedule of 25Gy in 5 fractions includes prescribed total doses between 24 and 26Gy. Episodes which have been created from multiple prescriptions may have sometimes contained different plans and, in these cases, the maximum prescribed dose fractionation per episode was taken.

Data completeness and consistency in the RTDS

In June, July, August, September and October 2020, several radiotherapy centres did not submit data into RTDS. This will have resulted in under-ascertainment of the true volume of rectal radiotherapy delivered in these months so adjustments were made. This was done by determining the average monthly proportion of episodes these Centres ordinarily contributed to the national rate of use rectal radiotherapy in 2019 and then uplifting the observed rates of radiotherapy use by this proportion.

Supplementary methods for subsequent analyses

Updates to the methods used for later analyses may be found at

http://www.ndph.ox.ac.uk/corectr/covid19

References for supplementary methods

1. NHS England. NHS Improvement. Cancer Waiting Times. 2020. https://www.england.nhs.uk/statistics/statistical-work-areas/cancer-waiting-times/ (accessed 17/10/2020 2020).
2. NHS England . Monthly diagnostic data. 2020. https://www.england.nhs.uk/statistics/statistical-work-areas/diagnostics-waiting-times-and-activity/monthly-diagnostics-waiting-times-and-activity/monthly-diagnostics-data-2020-21/ (accessed 28/10/20 2020).
3. NHS Digital. Secondary Uses Service (SUS). 2020. https://digital.nhs.uk/services/secondary-uses-service-sus (accessed 24/10/2020 2020).
4. Health and Social Care Information Centre. Hospital Episode Statistics. 2015. http://www.hscic.gov.uk/hes (accessed 27/10/2015 2015).
5. World Health Organisation. International Statistical Classification of Diseases and Related Health Problems: ICD10. Geneva: World Health Organisation; 2004.
6. NHS Digital. National clinical coding standards: OPCS-4 (2017), accurate data for quality information. Leeds: HMSO; 2017.

Table 1: International Classification of Disease Version 10 codes used to identify colorectal cancer related hospital admissions

Code	3-Digit Description	4-Digit Description
C180	Malignant neoplasm of colon	Caecum
C181	Malignant neoplasm of colon	Appendix
C182	Malignant neoplasm of colon	Ascending colon
C183	Malignant neoplasm of colon	Hepatic flexure
C184	Malignant neoplasm of colon	Transverse colon
C185	Malignant neoplasm of colon	Splenic flexure
C186	Malignant neoplasm of colon	Descending colon
C187	Malignant neoplasm of colon	Sigmoid colon
C188	Malignant neoplasm of colon	Overlapping lesion of colon
C189	Malignant neoplasm of colon	Colon, unspecified
C19X	Malignant neoplasm of rectosigmoid junction	
C20X	Malignant neoplasm of rectal	

Table 2: Office of Population Censuses Surveys Classification of Surgical Operations and Procedures 4th (OPCS4) revision codes used to define major resections and non-resectional surgery for colorectal cancer

Codes	3-Digit Description	4-Digit Description
H041	Total excision of colon and rectum	Panproctocolectomy and ileostomy
H042	Total excision of colon and rectum	Panproctocolectomy and anastomosis of ileum to anus and creation of pouch HFQ
H043	Total excision of colon and rectum	Panproctocolectomy and anastomosis of ileum to anus NEC
H048	Total excision of colon and rectum	Other specified total excision of colon and rectum
H049	Total excision of colon and rectum	Unspecified total excision of colon and rectum
H051	Total excision of colon	Total colectomy and anastomosis of ileum to rectum
H052	Total excision of colon	Total colectomy and ileostomy and creation of rectal fistula HFQ
H053	Total excision of colon	Total colectomy and ileostomy NEC
H058	Total excision of colon	Other specified total excision of colon
H059	Total excision of colon	Unspecified total excision of colon
H061	Extended excision of right hemicolon	Extended right hemicolectomy and end to end anastomosis
H062	Extended excision of right hemicolon	Extended right hemicolectomy and anastomosis of ileum to colon
H063	Extended excision of right hemicolon	Extended right hemicolectomy and anastomosis NEC
H064	Extended excision of right hemicolon	Extended right hemicolectomy and ileostomy HFQ
H065	Extended excision of right hemicolon	Extended right hemicolectomy and end to side anastomosis
H068	Extended excision of right hemicolon	Other specified extended excision of right hemicolon
H069	Extended excision of right hemicolon	Unspecified extended excision of right hemicolon
H071	Other excision of right hemicolon	Right hemicolectomy and end to end anastomosis of ileum to colon
H072	Other excision of right hemicolon	Right hemicolectomy and side to side anastomosis of ileum to transverse colon
H073	Other excision of right hemicolon	Right hemicolectomy and anastomosis NEC
H074	Other excision of right hemicolon	Right hemicolectomy and ileostomy HFQ
H075	Other excision of right hemicolon	Right hemicolectomy and end to side anastomosis
H078	Other excision of right hemicolon	Other specified other excision of right hemicolon
H079	Other excision of right hemicolon	Unspecified other excision of right hemicolon
H081	Excision of transverse colon	Transverse colectomy and end to end anastomosis
H082	Excision of transverse colon	Transverse colectomy and anastomosis of ileum to colon
H083	Excision of transverse colon	Transverse colectomy and anastomosis NEC
H084	Excision of transverse colon	Transverse colectomy and ileostomy HFQ

Codes	3-Digit Description	4-Digit Description
H085	Excision of transverse colon	Transverse colectomy and exteriorisation of bowel NEC
H086	Excision of transverse colon	Transverse colectomy and end to side anastomosis
H088	Excision of transverse colon	Other specified excision of transverse colon
H089	Excision of transverse colon	Unspecified excision of transverse colon
H091	Excision of left hemicolon	Left hemicolectomy and end to end anastomosis of colon to rectum
H092	Excision of left hemicolon	Left hemicolectomy and end to end anastomosis of colon to colon
H093	Excision of left hemicolon	Left hemicolectomy and anastomosis NEC
H094	Excision of left hemicolon	Left hemicolectomy and ileostomy HFQ
H095	Excision of left hemicolon	Left hemicolectomy and exteriorisation of bowel NEC
H096	Excision of left hemicolon	Left hemicolectomy and end to side anastomosis
H098	Excision of left hemicolon	Other specified excision of left hemicolon
H099	Excision of left hemicolon	Unspecified excision of left hemicolon
H101	Excision of sigmoid colon	Sigmoid colectomy and end to end anastomosis of ileum to rectum
H102	Excision of sigmoid colon	Sigmoid colectomy and anastomosis of colon to rectum
H103	Excision of sigmoid colon	Sigmoid colectomy and anastomosis NEC
H104	Excision of sigmoid colon	Sigmoid colectomy and ileostomy HFQ
H105	Excision of sigmoid colon	Sigmoid colectomy and exteriorisation of bowel NEC
H106	Excision of sigmoid colon	Sigmoid colectomy and end to side anastomosis
H108	Excision of sigmoid colon	Other specified excision of sigmoid colon
H109	Excision of sigmoid colon	Unspecified excision of sigmoid colon
H111	Other excision of colon	Colectomy and end to end anastomosis of colon to colon NEC
H112	Other excision of colon	Colectomy and side to side anastomosis of ileum to colon NEC
H113	Other excision of colon	Colectomy and anastomosis NEC
H114	Other excision of colon	Colectomy and ileostomy NEC
H115	Other excision of colon	Colectomy and exteriorisation of bowel NEC
H116	Other excision of colon	Colectomy and end to side anastomosis NEC
H118	Other excision of colon	Other specified other excision of colon
H119	Other excision of colon	Unspecified other excision of colon
H291	Subtotal excision of colon	Subtotal excision of colon and rectum and creation of colonic pouch and anastomosis of colon to anus
H292	Subtotal excision of colon	Subtotal excision of colon and rectum and creation of colonic pouch NEC
H293	Subtotal excision of colon	Subtotal excision of colon and creation of colonic pouch and anastomosis of colon to rectum
H294	Subtotal excision of colon	Subtotal excision of colon and creation of colonic pouch NEC
H298	Subtotal excision of colon	Other specified subtotal excision of colon
H299	Subtotal excision of colon	Unspecified subtotal excision of colon
H308	Other operations on colon	Other specified other operations on colon
H309	Other operations on colon	Unspecified other operations on colon
H331	Excision of rectum	Abdominoperineal excision of rectum and end colostomy
H332	Excision of rectum	Proctectomy and anastomosis of colon to anus
H333	Excision of rectum	Anterior resection of rectum and anastomosis of colon to rectum using staples
H334	Excision of rectum	Anterior resection of rectum and anastomosis NEC
H335	Excision of rectum	Rectosigmoidectomy and closure of rectal stump and exteriorisation of bowel
H336	Excision of rectum	Anterior resection of rectum and exteriorisation of bowel
H337	Excision of rectum	Perineal resection of rectum HFQ
H338	Excision of rectum	Other specified excision of rectum

Codes	3-Digit Description	4-Digit Description
H339	Excision of rectum	Unspecified excision of rectum
H404	Operations on rectum through anal sphincter	Trans-sphincteric anastomosis of colon to anus
H411	Other operations on rectum through anus	Rectosigmoidectomy and peranal anastomosis
H414	Other operations on rectum through anus	Peranal mucosal proctectomy and endoanal anastomosis
H415	Other operations on rectum through anus	Peranal resection of rectum using staples
H418	Other operations on rectum through anus	Other specified other operations on rectum through anus
H419	Other operations on rectum through anus	Unspecified other operations on rectum through anus
X141	Clearance of pelvis	Total exenteration of pelvis
X142	Clearance of pelvis	Anterior exenteration of pelvis
X143	Clearance of pelvis	Posterior exenteration of pelvis
X148	Clearance of pelvis	Other specified clearance of pelvis
X149	Clearance of pelvis	Unspecified clearance of pelvis

Table 3: Office of Population Censuses Surveys Classification of Surgical Operations and Procedures 4th revision (OPCS4) codes used to define laparoscopic procedures

OPCS4 Code	OPSC4 Group	OPSC4 Description
Y502	Approach through abdominal cavity	Laparotomy approach NEC
Y508	Approach through abdominal cavity	Other specified approach through abdominal cavity
Y509	Approach through abdominal cavity	Unspecified approach through abdominal cavity
Y714	Late operations NOC	Failed minimal access approach converted to open
Y751	Minimal access to abdominal cavity	Laparoscopically assisted approach to abdominal cavity
Y752	Minimal access to abdominal cavity	Laparoscopic approach to abdominal cavity NEC
Y753	Minimal access to abdominal cavity	Robotic minimal access approach to abdominal cavity
Y754	Minimal access to abdominal cavity	Hand assisted minimal access approach to abdominal cavity
Y755	Minimal access to abdominal cavity	Laparoscopic ultrasonic approach to abdominal cavity
Y758	Minimal access to abdominal cavity	Other specified minimal access to abdominal cavity
Y759	Minimal access to abdominal cavity	Unspecified minimal access to abdominal cavity

Table 4: OPCS4 codes indicating major resections associated with the opening of an ileostomy/stoma

OPCS4 code	OPSC4 Group	OPCS4 Description
H041	Total excision of colon and rectum	Panproctocolectomy and ileostomy
H052	Total excision of colon	Total colectomy and ileostomy and creation of rectal fistula HFQ
H053	Total excision of colon	Total colectomy and ileostomy NEC
H064	Extended excision of right hemicolon	Extended right hemicolectomy and ileostomy HFQ
H074	Other excision of right hemicolon	Right hemicolectomy and ileostomy HFQ
H084	Excision of transverse colon	Transverse colectomy and ileostomy HFQ
H085	Excision of transverse colon	Transverse colectomy and exteriorisation of bowel NEC
H094	Excision of left hemicolon	Left hemicolectomy and ileostomy HFQ
H095	Excision of left hemicolon	Left hemicolectomy and exteriorisation of bowel NEC
H104	Excision of sigmoid colon	Sigmoid colectomy and ileostomy HFQ
H105	Excision of sigmoid colon	Sigmoid colectomy and exteriorisation of bowel NEC
H114	Other excision of colon	Colectomy and ileostomy NEC
H115	Other excision of colon	Colectomy and exteriorisation of bowel NEC

OPCS4 code	OPSC4 Group	OPCS4 Description
H331	Excision of rectum	Abdominoperineal excision of rectum and end colostomy
H335	Excision of rectum	Rectosigmoidectomy and closure of rectal stump and exteriorisation of bowel
H336	Excision of rectum	Anterior resection of rectum and exteriorisation of bowel

Table 5: OPCS4 codes indicating the opening of a stoma

OPCS4 code	OPSC4 group	OPSC4 Description
H141	Exteriorisation of caecum	Tube caecostomy
H144	Exteriorisation of caecum	Appendicocaecostomy
H148	Exteriorisation of caecum	Other specified exteriorisation of caecum
H149	Exteriorisation of caecum	Unspecified exteriorisation of caecum
H151	Other exteriorisation of colon	Loop colostomy
H152	Other exteriorisation of colon	End colostomy
H157	Other exteriorisation of colon	Percutaneous endoscopic sigmoid colostomy
H158	Other exteriorisation of colon	Other specified other exteriorisation of colon
H159	Other exteriorisation of colon	Unspecified other exteriorisation of colon
G741	Creation of artificial opening into ileum	Creation of continent ileostomy
G742	Creation of artificial opening into ileum	Creation of temporary ileostomy
G743	Creation of artificial opening into ileum	Creation of defunctioning ileostomy
G748	Creation of artificial opening into ileum	Other specified creation of artificial opening into ileum
G749	Creation of artificial opening into ileum	Unspecified creation of artificial opening into ileum
H328	Exteriorisation of colon	Other specified exteriorisation of colon
H329	Exteriorisation of colon	Unspecified exteriorisation of colon

Supplementary Results

Supplementary Table 1: Monthly number and percent reduction in colorectal cancer operations, by type

	Operations		Laparoscopic operations		Stoma forming operations		Operations following an emergency admission	
Time period	N*	Percent reduction (95%CI)	N*	Percent reduction (95%CI)	N*	Percent reduction (95%CI)	N*	Percent reduction (95%CI)
2019 Monthly average	2,003		1,175		889		391	
Jan-20	2,245	-12 (-28,2)	1,376	-17 (-37,0)	1,016	-14 (-30,-1)	393	0 (-17,14)
Feb-20	1,979	1 (-14,14)	1,193	-2 (-20,14)	865	3 (-11,15)	348	11 (-5,25)
Mar-20	2,129	-6 (-22,7)	1,246	-6 (-25,10)	942	-6 (-21,7)	350	11 (-5,24)
Apr-20	1,378	31 (19,42)	341	71 (61,78)	773	13 (0,25)	304	22 (8,35)
May-20	1,339	33 (21,43)	460	61 (49,70)	741	17 (4,28)	374	5 (-12,19)
Jun-20	1,576	21 (8,33)	683	42 (28,53)	844	5 (-9,17)	450	-15 (-33,1)
Jul-20	1,712	15 (1,26)	870	26 (10,39)	886	0 (-14,13)	434	-11 (-28,5)
Aug-20	1,662	17 (3,29)	837	29 (13,41)	858	3 (-11,16)	461	-18 (-36,-2)
Sep-20	1,913	4 (-10,17)	1,125	4 (-14,19)	936	-5 (-20,8)	414	-6 (-23,9)
Oct-20	1,859	7 (-7,20)	1,131	4 (-14,19)	845	5 (-9,17)	388	1 (-16,15)
Apr-Oct-20 monthly average	1,634	18 (13,24)	778	34 (28,39)	841	5 (-1,11)	403	-3 (-11,4)

*Numbers in 2020 are adjusted using the methods described (Methods/Supplementary methods) for any reduction in submission of data

Supplementary Table 2: Monthly number and percent reduction in colon cancer operations, by type

	Operations		Laparoscopic operations		Stoma forming operations		Operations following an emergency admission	
Time period	N*	Percent reduction (95%CI)	N*	Percent reduction (95%CI)	N*	Percent reduction (95%CI)	N*	Percent reduction (95%CI)
2019 Monthly average	1,296		786		325		306	
Jan-20	1,469	-13 (-30,1)	937	-19 (-42,0)	373	-15 (-29,-2)	305	0 (-16,15)
Feb-20	1,314	-1 (-17,12)	807	-3 (-24,15)	337	-4 (-17,8)	301	2 (-15,16)
Mar-20	1,397	-8 (-24,6)	829	-6 (-27,12)	362	-11 (-25,1)	277	10 (-6,23)
Apr-20	900	31 (17,42)	221	72 (60,80)	376	-16 (-30,-3)	244	20 (6,33)
May-20	892	31 (18,42)	313	60 (47,70)	353	-8 (-22,4)	298	3 (-14,17)
Jun-20	1,020	21 (7,33)	453	42 (26,55)	358	-10 (-24,2)	365	-19 (-37,-3)
Jul-20	1,062	18 (4,30)	549	30 (13,44)	316	3 (-10,14)	340	-11 (-29,4)
Aug-20	1,063	18 (4,30)	552	30 (12,44)	341	-5 (-19,7)	365	-19 (-37,-3)
Sep-20	1,248	4 (-12,17)	758	4 (-17,20)	364	-12 (-26,0)	338	-10 (-28,5)
Oct-20	1,273	2 (-14,15)	811	-3 (-25,14)	349	-7 (-21,5)	308	0 (-17,14)
Apr-Oct-20 monthly average	1,065	18 (12,23)	523	33 (27,40)	351	-8 (-14,-2)	323	-5 (-13,2)

*Numbers in 2020 are adjusted using the methods described (Methods/Supplementary methods) for any reduction in submission of data

Supplementary Table 3: Monthly number and percent reduction in rectal cancer operations, by type

	Operations		Laparoscopic operations		Stoma forming operations		Operations following an emergency admission	
Time period	N*	Percent reduction (95%CI)	N*	Percent reduction (95%CI)	N*	Percent reduction (95%CI)	N*	Percent reduction (95%CI)
2019 Monthly average	707		389		564		85	
Jan-20	776	-10 (-28,6)	439	-13 (-35,6)	643	-14 (-33,2)	88	-3 (-26,15)
Feb-20	665	6 (-11,20)	386	1 (-20,18)	528	6 (-11,21)	47	45 (28,58)
Mar-20	732	-3 (-21,11)	417	-7 (-28,11)	581	-3 (-21,12)	73	15 (-6,31)
Apr-20	478	32 (18,44)	120	69 (57,78)	398	29 (15,42)	60	30 (11,45)
May-20	447	37 (23,48)	147	62 (49,72)	388	31 (17,43)	76	11 (-10,28)
Jun-20	556	21 (6,34)	230	41 (25,54)	486	14 (-3,28)	85	0 (-22,19)
Jul-20	650	8 (-8,22)	321	18 (-1,33)	570	-1 (-19,14)	93	-10 (-33,10)
Aug-20	599	15 (-1,29)	284	27 (9,41)	517	8 (-8,23)	96	-13 (-36,7)
Sep-20	665	6 (-11,20)	368	6 (-14,22)	572	-1 (-19,14)	77	10 (-11,27)
Oct-20	586	17 (1,30)	320	18 (-1,33)	496	12 (-4,26)	80	6 (-16,24)
Apr-Oct-20 monthly average	879	-24 (-33,-16)	256	34 (28,40)	489	13 (6,20)	81	5 (-4,13)

*Numbers in 2020 are adjusted using the methods described (Methods/Supplementary methods) for any reduction in submission of data

Supplementary Figure 1: Monthly number and proportion of operations for colorectal, colonic and rectal cancer that were undertaken laparoscopically

The mean monthly number (and +/- one standard deviation (SD)) of laparoscopic operations in 2019 is plotted alongside the monthly counts for both 2019 and 2020 in connected line graphs in which vertical lines represent 1 SD for monthly counts or proportions. The 2020 rate is based on counts adjusted for incomplete coding (see methods/supplementary methods).

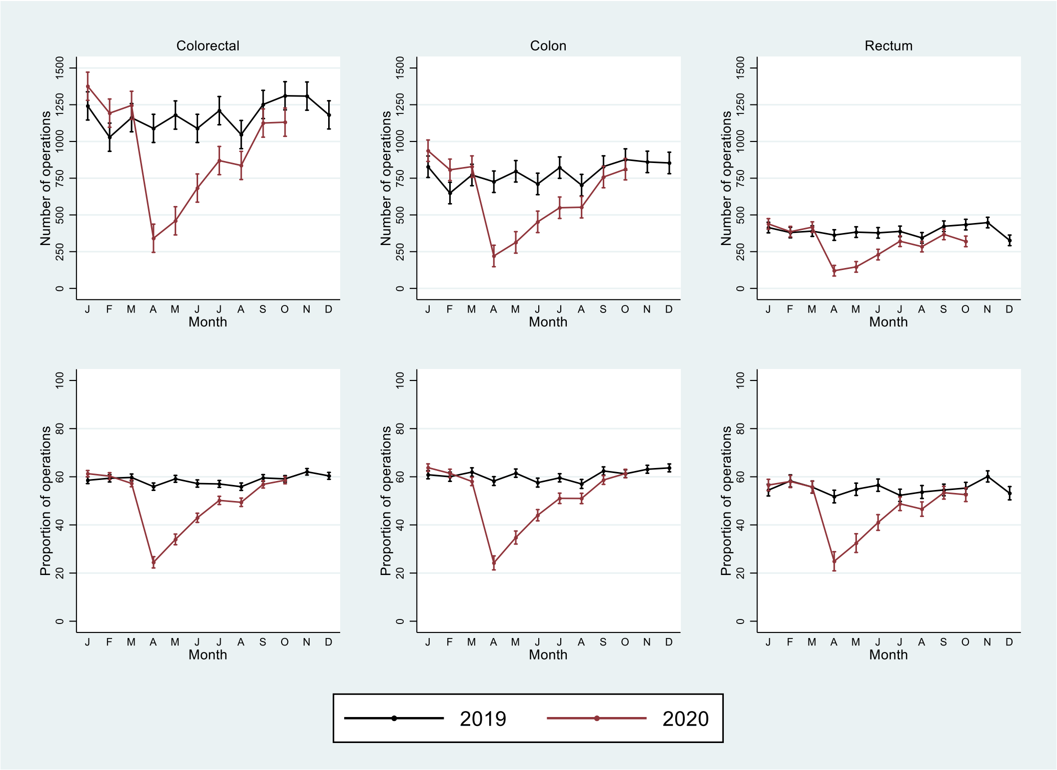

Supplementary Figure 2: Monthly number and proportion of operations for colorectal, colonic and rectal cancer that led to the formation of a stoma

Symbols and conventions as in Supplementary Figure 1. The 2020 rate is based on counts adjusted for incomplete coding (see methods/supplementary methods).

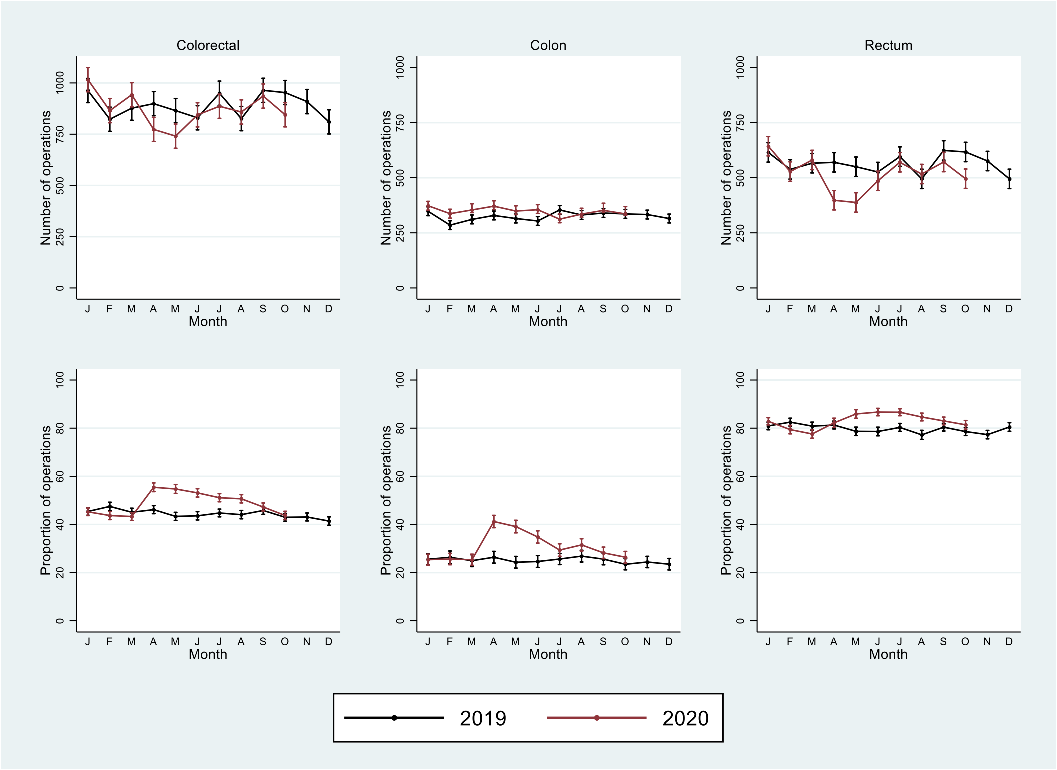

Supplementary Figure 3: Monthly number and proportion of operations for colorectal, colonic and rectal cancer that were undertaken following an emergency admission

Symbols and conventions as in Supplementary Figure 1. The 2020 rate is based on counts adjusted for incomplete coding (see methods/supplementary methods).

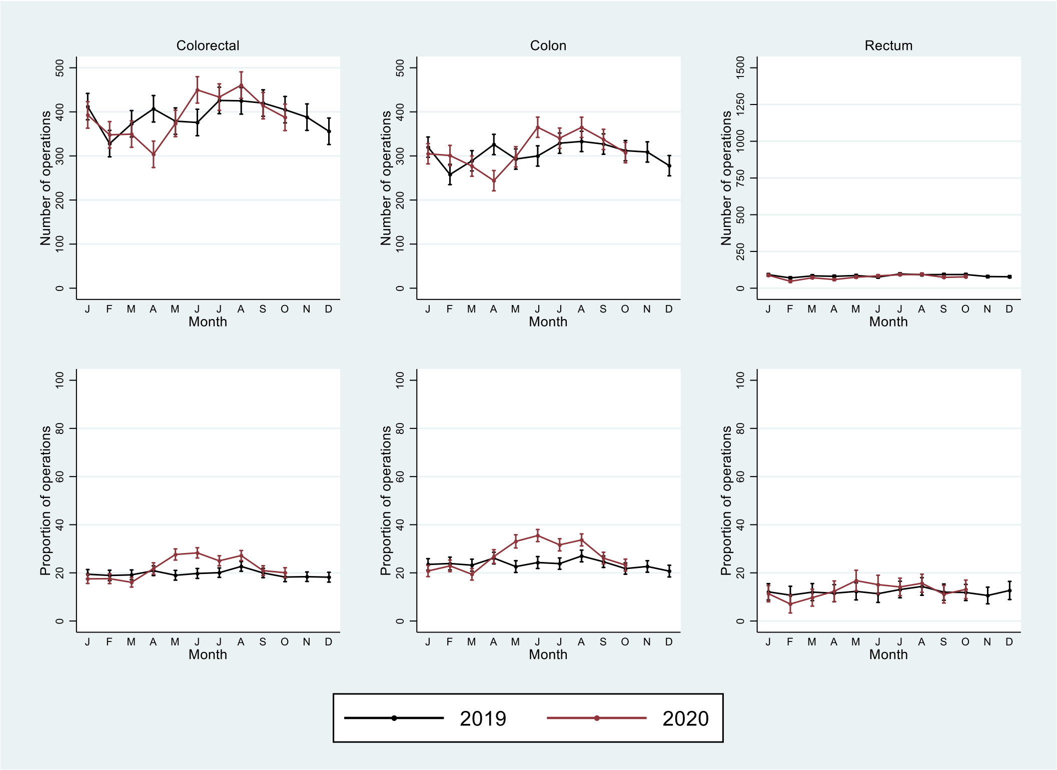

Supplement: Supplementary appendix [file mmc1.pdf]
